# Combination of chemotherapy and immunotherapy may overcome the resistance to immunotherapy alone in pulmonary lymphoepithelial carcinoma

**DOI:** 10.1002/kjm2.12827

**Published:** 2024-04-09

**Authors:** Huei‐Yang Hung, Wei‐An Lai, Cheng‐Hao Chuang, Chih‐Jen Yang

**Affiliations:** ^1^ Division of Pulmonary and Critical Care Medicine, Department of Internal Medicine Kaohsiung Medical University Hospital, Kaohsiung Medical University Kaohsiung Taiwan; ^2^ Department of Pathology Kaohsiung Medical University Hospital, Kaohsiung Medical University Kaohsiung Taiwan; ^3^ School of Post‐Baccalaureate Medicine, College of Medicine, Kaohsiung Medical University Kaohsiung Taiwan

Dear Editor,

Pulmonary lymphoepithelial carcinoma (PLEC) is a rare and challenging subtype of non‐small cell lung cancer (NSCLC), accounting for only 0.25%–0.9% of all NSCLC cases.^1^ Due to its rarity, there are currently no established standardized treatment protocols for PLEC, leading physicians to often rely on multimodal treatment strategies, especially in advanced‐stage cases.^1^, ^2^ This treatment typically includes a combination of chemotherapy, radiotherapy, and immunotherapy.

We present a compelling case of a 71‐year‐old nonsmoking Taiwanese woman with dementia who was diagnosed with PLEC. She initially presented with a chronic cough, which led to the discovery of an 11.4 cm mass in her left upper lung (Figure 1A), extending to the mediastinum and accompanied by metastases and pleural effusion. Biopsies confirmed lymphoepithelial carcinoma, and a computed tomography scan revealed clinical stage IVA. Immunohistochemical tests were positive for p40, and in situ hybridization for Epstein–Barr encoded region revealed positive neoplastic cell nuclei (magnification ×200; Figure 1B) with a high expression of programmed death‐ligand 1 (PD‐L1) of up to 90% in the tumor cells. Notably, there were no significant genomic alterations, including in the epidermal growth factor receptor, anaplastic lymphoma kinase, or reactive oxygen species‐1 genes.

**FIGURE 1:**
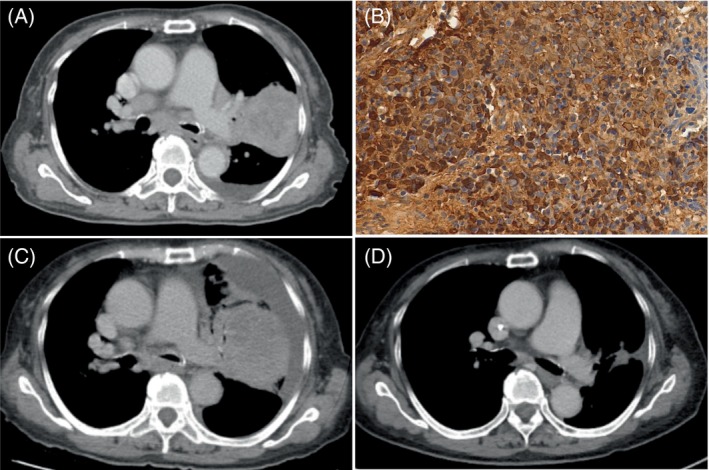
(A) Chest CT showing an 11.4 cm mass in the left upper lung, indicative of stage IVA disease. (B) Epstein–Barr encoded region in situ hybridization, highlighting positive tumor cell nuclei (200× magnification). (C) Progression of the disease after 8 months and 12 cycles of treatment, evidenced by increased mass size and pleural effusion. (D) Marked reduction in tumor size and pleural effusion following four cycles of gemcitabine, carboplatin, and pembrolizumab chemotherapy. CT, computed tomography.

Initially, she was treated with pembrolizumab 200 mg alone every 21 days. After four cycles, the tumor size decreased. However, 8 months and 12 cycles later, the disease recurred, marked by an increase in tumor size and pleural effusion (Figure 1C). This led to a shift in treatment strategy, and chemotherapy with gemcitabine and carboplatin was introduced alongside a reduced dose of pembrolizumab (100 mg). Remarkably, there was a significant resolution in tumor size and pleural effusion after four cycles (Figure 1D), indicating a successful response to the new treatment regimen.

This case is particularly instructive in understanding the dynamics of treating PLEC.

Epstein–Barr virus (EBV) infection is linked to an increase in PD‐L1 expression in malignancies, and it is a critical factor in PLEC, with over 74% of PLEC cases showing positive PD‐L1 expression.^1^, ^2^ This high expression rate makes immune checkpoint inhibitors (ICIs) such as pembrolizumab a viable treatment option.^2^, ^3^ However, as this case illustrates, when initial immunotherapy is not entirely effective, adding chemotherapy can significantly enhance the treatment's effectiveness.

The synergistic effect observed in this patient can be attributed to the role of chemotherapy in releasing potentially immunogenic tumor antigens, thereby boosting the immune response against the tumor. This phenomenon has also been noted in other cases and studies. For example, a retrospective cohort study by Pang et al. involving 45 PLEC patients treated with ICIs found that combining chemotherapy with immunotherapy resulted in better progression‐free survival compared to ICI monotherapy.^4^ In addition, Tang et al. described a PLEC case that initially resisted first‐line chemotherapy and second‐line immunotherapy with nivolumab.^5^ The tumor was eventually controlled by incorporating additional chemotherapy into the treatment regimen.

In summary, this case highlights the potential of combining chemotherapy with immunotherapy in treating PLEC, particularly in instances where there is resistance to initial immunotherapy. The dramatic response observed in this patient after integrating chemotherapy underscores the need for further research and clinical trials. Such studies are essential to confirm the safety, efficacy, and optimal combination of treatments, including ICIs, in managing PLEC. As PLEC remains a rare and complex subtype of NSCLC, continued exploration of treatment strategies is crucial for improving patient outcomes in this challenging field.

## CONFLICT OF INTEREST STATEMENT

The authors declare no conflict of interest.
